# Molecular signatures induced by interleukin-2 on peripheral blood mononuclear cells and T cell subsets

**DOI:** 10.1186/1479-5876-4-26

**Published:** 2006-06-28

**Authors:** Ping Jin, Ena Wang, Maurizio Provenzano, Sara Deola, Silvia Selleri, Jiaqiang Ren, Sonia Voiculescu, David Stroncek, Monica C Panelli, Francesco M Marincola

**Affiliations:** 1 Immunogenetics Section, Department of Transfusion Medicine, Clinical Center, National Institutes of Health, Bethesda, Maryland, 20892, USA; 2 Immune Oncology Section, Department of Surgery, University Hospital ZLF, Hebelstrasse 20, 4031, Basel, Switzerland

## Abstract

Experimentally, interleukin-2 (IL-2) exerts complex immunological functions promoting the proliferation, survival and activation of T cells on one hand and inducing immune regulatory mechanisms on the other. This complexity results from a cross talk among immune cells which sways the effects of IL-2 according to the experimental or clinical condition tested. Recombinant IL-2 (rIL-2) stimulation of peripheral blood mononuclear cells (PBMC) from 47 donors of different genetic background induced generalized T cell activation and anti-apoptotic effects. Most effects were dependent upon interactions among immune cells. Specialized functions of CD4 and CD8 T cells were less dependent upon and often dampened by the presence of other PBMC populations. In particular, cytotoxic T cell effector function was variably affected with a component strictly dependent upon the direct stimulation of CD8 T cells in the absence of other PBMC. This observation may provide a roadmap for the interpretation of the discrepant biological activities of rIL-2 observed in distinct pathological conditions or treatment modalities.

## 

Human recombinant interleukin (rIL)-2 is a cytokine approved by the Food and Drug Administration for the treatment of advanced melanoma and renal cell cancer because it can induce complete cancer regression in a small but consistent proportion of patients [1,2]. In addition, systemic rIL-2 alone [3] or in combination with antigen-specific immunization [4] increases the frequency of interferon (IFN)-γ-producing memory T cells in human immunodeficiency virus-infected individuals and improves anti-viral responses [5]. Conversely, IL-2 receptor (IL-2R) antagonists are used to decrease the frequency of allograft rejection [6]. Thus, rIL-2 is used clinically to generate desired immune responses and its blockade to hamper unwanted ones. Recently, however, it has been suggested that rIL-2 increases the frequency of regulatory T cells in cancer patients [7]. Concordantly, while lymph depletion, believed to preferentially eliminate regulatory T cells, restores the effectiveness of rIL-2 administered together with tumor-specific T cells to patients with rIL-2-refractory melanoma [8]. Empirically, *in vivo *rIL-2 administration induces high serum levels of acute phase reactants [9,10]. and a broad range of cytokines with conflicting pro- or anti-inflammatory properties [11] whose secretion is only partially accountable to direct interactions between rIL-2 and IL-2R-bering cells [12]. Thus, it should not surprise that rIL-2 may display a bipolar behavior in complex systems inducing opposite effects depending upon the circumstances in which it is delivered sometimes inducing activation, proliferation and survival of cytotoxic T and natural killer cells [13,14] and other times inducing tolerance through expansion of regulatory T cells [15] or activation-induced cell death [16].

Sorting the biological component relevant to tumor rejection out of the broad effects induced by rIL-2 administration has important practical implications because some are responsible for severe toxicity limiting its clinical usefulness [17-20]. A number of clinical and experimental observations suggest that rIL-2-induced toxicity is partly linked to its effectiveness since blocking toxicity abrogates efficacy [21,22]. Yet, the biological mechanism(s) responsible for cancer rejection diverge(s) at some point from toxicity since the latter is characterized by an idiosyncratic and unpredictable pattern statistically dissociated from clinical outcome [23].

We have previously compared the systemic effects of rIL-2 in peripheral blood monocytes (PBMC) to the peripheral effects induced in melanoma metastases three hours after administration to patient with metastatic melanoma [24]. Consistent peripheral effects were observed which were distinct from the systemic effects and included activation of tumor-associated macrophages and up-regulation of interferon stimulated genes (ISGs) with minimal induction of migration, activation and proliferation of T cells. These effects were insufficient to induce cancer regression since they occurred in all the metastases independently of clinical outcome. However, analysis of a metastasis that regressed in response to therapy suggested that recruitment of cytotoxic T cells and the activation of their effector function at the tumor site is required for cancer rejection [24]. Indeed, *in vitro *activation of tumor antigen-specific T cell reproduced transcriptional signatures consistent with the activation of effector functions associated with tumor rejection [25]. Thus, it appears that peripherally rIL-2 induces two transcriptional patterns: a reproducible activation ISGs which is unrelated to cancer rejection and an occasional induction of T cell effector functions closely linked to cancer clearance. These different outcomes might be determined by a distinct immune environment in responsive compared to refractory tumors resulting from alternative interactions among different immune cells [26].

To provide a road map for the interpretation of future clinical studies, we characterized molecular pathways associated with rIL-2 by stimulating *in vitro *PBMC that most likely approximate the immunological cross talk occurring *in vivo *following rIL-2 administration [24] and sorted direct effects on CD4 and CD8-expressing T cells from those resulting through a concerted cross talk among PBMC. PBMC obtained by leukapheresis from 47 normal donors of distinct ethnic background (30 Caucasian and 17 Chinese subjects) were stimulated *in vitro *with 300 IU/ml of rIL-2. By selecting two ethnic backgrounds, homogeneity of results due to sample bias was minimized increasing, at the same time, the chances of identifying genetic traits relevant to IL-2 biology. The following known functions of rIL-2 were considered: 1) modulation of signaling down-stream of the IL-2R; 2) amplification of rIL-2 signaling through enhancement of IL-2R complex affinity/availability and other receptors; 3) Modulation of signaling through the T cell receptor (TCR); 4) Induction of lymphokine and immune-effector molecules; 5) T cell subsets-specific effects. Predominantly we followed signaling pathways previously characterized by others [27-29]. The results indicate that rIL-2 induces generalized T cell activation and anti-apoptotic effects, which are highly dependent on bystander interactions among immune cells other than T cells while specialized function of CD4 and CD8 T cells are less dependent on them. Moreover, the effector function of cytotoxic T cells follows a bipolar pattern variably affected by the presence of bystander immune cells with a fraction strictly dependent upon the direct stimulation of CD8 T cells by rIL-2 in the absence of other PBMC.

## Results

### Global effects of rIL-2 on the transcriptional pattern of PBMC

The present study was designed to comprehensively characterize molecular pathways induced in PBMC by the exogenous administration of human rIL-2 at a concentration corresponding to intermediate pharmaceutical doses (300 IU/ml) [24]. Donors from two biogeographically-defined backgrounds (30 Caucasians and 17 Chinese) [30] were selected to decrease sampling-dependent biases. This collection of donor PBMC is part of a larger study aimed at the comparison of immunological and genetic traits in human subjects of different genetic background (NIH protocol 04-CC-0007).

We first analyzed the effects of rIL-2 on PBMC from all donors (Table 1). A two-tailed paired *t *test (cut-off p_2_-value < 0.005) comparing gene expression changes between non-treated and rIL-2-treated PBMC from the 47 donors identified 1,690 cDNA clones (complete list available as additional file 1). To determine whether the number of genes found to be differentially expressed was higher than expected by chance, we applied a permutation test randomly shifting the assignment of samples among different classes and re-computed the *t *test statistics each time. This analysis was repeated 10,000 times and the proportion of the random replications that resulted in as many significant genes as seen in the actual data set was reported as the significance level. Multivariate and univariate permutation test supported the significance of the findings (p_2_-value = 0 and 0.007 respectively). The same pair wise analysis was performed within each ethnic group and identified 1,340 cDNA clones in Caucasian and 411 in Chinese samples. To correct for the different number of donors included in each ethnic group the same analysis was performed testing PBMC from the first 17 consecutive Caucasian donors. Even in this case, a differential expression of a larger number of genes (784 genes) was observed in Caucasians. In all cases multivariate and univariate permutation analyses were significant.

**Table 1:** 

**Ethnic Group**	**Class Comparison**	**Student t test** **p_2_-value < 0.005** **# genes differentially expressed**	**Permutation Test (p-value)**	
			**Multivariate **	**Univariate**
*a*) All Donors (n = 47)	IL-2 vs No-Stim	1690	**0**	**0.007**
*b*) Chinese (n = 17)	IL-2 vs No-Stim	411	**0.001**	**0.019**
*c*) Caucasians (n = 30)	IL-2 vs No-Stim	1340	**0**	**0.009**
*d*) Caucasians (n = 17)	IL-2 vs No-Stim	784	**0**	**0.01**

				

***Ethnic Group***	***Class Comparison***	***Student t test*** ***p_2_-value < 0.05*** ***# genes differentially expressed***	***Permutation Test (p-value)***	
			***Multivariate ***	***Univariate***

*e*) All Donors (n = 47)	IL-2 vs No-Stim	3926	**0**	**0**

Genes with expression altered by exposure of PBMC in response to rIL-2 (300 IU/ml) *in vitro *– BMC were obtained from 30 Caucasian (Ca) and 17 Chinese (Ch) normal donors. Class comparison was performed by two-tailed Student *t *test with as cutoff of significance a p_2_-value <0.005 comparing CY5/Cy3 log_2 _ratios between test and reference samples (see Materials and Methods). Multivariate and univariate permutation test evaluated the significance of the comparisons.

Although the previous analysis suggested higher reactivity to rIL-2 in PBMC from Caucasians compared to Chinese, further analyses failed to identify convincingly significant differences among the two ethnic groups. In addition, sequencing of the IL-2Rβ chain did not support a genetically determined variability in the response to rIL-2. Of 8 polymorphic sites identified (one previously unreported at nucleotide 1283) only one lead to an amino acid substitution in position 391 ( additional file 2). Although this variant is potentially relevant because it is just proximal to a tyrosine residue (position 392) [14], it was functionally conservative (from an aspartic (asp) to a glutamic acid (glu) and was never present in homozygous conditions. Nine of 30 (30%) Caucasian and 9 of 17 (53%) Chinese subjects were heterozygous for both variants (Fisher's exact test p_2_-value = 0.2). The heterozygous phenotype did not significantly affect the transcriptional program of PBMC (data not shown) suggesting that the response to rIL-2 is conserved among different ethnic groups and it is not affected by polymorphisms of the signaling "work horse" of the IL-2R [31]. Thus, contrary to other cytokines [32], the response to rIL-2 does not seem to be strongly influenced by genetic background at least at the population level. The subsequent analysis was, therefore, based on the complete data set. A second more inclusive analysis was also performed with a less stringent threshold of significance (paired two-tailed Student *t *test p_2_-value < 0.05, **analysis *e***, Table 1). The analysis identified 3,926 cDNA clones of which 1,973 (corresponding to 1,592 named genes) were up regulated and 1,953 down regulated (1,491 named genes) by rIL-2. This second analysis was used to include genes of borderline significance when relevant for purposes of discussion but did not serve as the primary data base.

#### 1) Modulation of signaling down-stream of the interleukin-2 receptor

**Figure 1:**
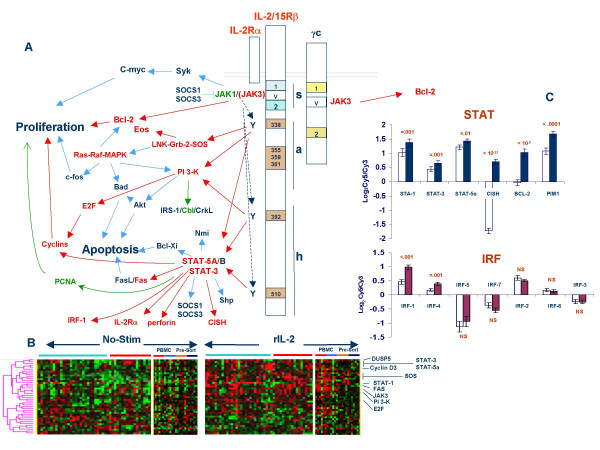
**A **– Signaling pathways linked to the intracellular domains of the IL-2Rβ and the γc chains based on Gaffen SL [14] and Leonard WJ and O'Shea [120]. Highlighted boxes refer to different domains of the receptor subunits. Red arrows refer to pathways that appeared activated by rIL-2 administration and red gene names refer to those significantly up-regulated by rIL-2. Blue arrows and names refer respectively to pathways and genes whose expression was not affected by rIL-2. In green are pathways and genes significantly down regulated by rIL-2. The letters "s", "a" and "h" refer to the proximal, middle and distal regions of the IL-2Rβ chain – **B **Relative expression of various genes associated with IL-2R signaling whose expression is significantly affected by rIL-2 based on a two-tailed paired *t *test comparing non-stimulated with rIL-2-stiulated PBMC from all 47 donors. Ratios are displayed according to the central method for normalization [92]. Light blue and red horizontal bares underline PBMC sample from Caucasian and Chinese donors; in separate panels CD4 (small red horizontal bar) and CD8 T cells (light blue bar) separated by negative bead separation (Miltenyi Biotech, Bergisch Gladbach, Germany) are compared with CD4 and CD8 T cells (orange and dark blue horizontal bars respectively) purified before exposure to rIL-2 – **C **Top, panel; mRNA expression (expressed as log_2 _CY5/Cy3) of signal transducers and activators of transcription (STAT)-1, -3 and 5a in non-stimulated PBMC (white bars) or after stimulation with 300 IU/ml of rIL-2 (Blue filled bars). In addition, proportional mRNA levels of three well characterized targets of STAT-5 are shown (CISH, BCL-2 and PIM1); bottom panel: mRNA expression of interferon regulatory factors (IRF) in non-stimulated PBMC (white bars) or after stimulation with 300 IU/ml of rIL-2 (maroon filled bars). Significance is based on a paired two-tailed Student *t *test between non-stimulated and rIL-2 stimulated samples from all 47 donors (**analysis *a*, **table 1).

This analysis was based on a schematic representation by Gaffen SL [31] of the signaling domains of the IL-2R separating pathways associated with the IL-2/15Rβ cytoplasmic tail from those associated with the common γ chain (γc) cytoplasmic tail (Figure 1A and 1B).

##### Pathways associated with the IL-2/15Rβ cytoplasmic tail

The IL-2/15Rβ cytoplasmic tail is the work horse of IL-2 signaling [31]. The proximal cytoplasmic tail ("s" region) contains a serine rich domain tightly associated with signaling through Janus (JAK) kinases 1 and (upon receptor activation) JAK-3 [33,34]. JAK-3 was significantly up-regulated by rIL-2 (paired *t *test p_2_-value < 0.001) while JAK-1 was significantly down-regulated (paired *t *test p_2_-value < 0.005). The central cytoplasmic region ("a" region) includes four tyrosine residues whose phosphorilation is associated with signaling events. The motif surrounding Tyr 338 is a docking site for Shc [35] which connects signaling to the Ras-Raf-MAPK pathway. Sch expression was not altered by rIL-2; however, several genes associated with its function were strongly up-regulated including Grb-2, SOS, LNK [36] and EOS (ZNFN1A4)-Bcl-2 (paired *t *test p_2_-values < 0.001 for all). In addition, rIL-2 had no effect on the expression of the MAPK-dependent gene Bad (Bcl-2 antagonist of cell death) and no effect on the transcription of c-fos while it significantly up-regulated the catalytic peptides of the phosphatidylinositol 3-kinase (PI 3-K) including the α, β and δ subunits. This may represent a central mechanism through which IL-2 promotes survival of T cells [37,38]. In association, E2F was found to be significantly up regulated and its up regulation corresponded to increased expression of various cyclins with a predominant effect on cyclin D consistent with previous reports [39].

An important pathway regulated by tyrosine containing domains (both in the "a" and the "h" region) is the phosphorilation of signal transducer and activator of transcription (STAT)-3 and STAT-5A and -B. STAT-3 and 5A were significantly induced by rIL-2 (Figure 1C) and their expression tightly correlated with that of the IL-2Rα chain expression as previously described [40-44]. On the contrary, the expression of the homologous genes STAT-5B was not significantly affected. This, however, did not affect STAT-5 dependent transcription at least at this time point since the expression of three well-defined target genes of STAT-5 (*CISH, BCL-2 *and *PIM1*) involved in survival, proliferation and negative regulation of cytokine receptor signaling was strongly up-regulated by rIL-2 [45]. Downstream of STAT-5 various cyclins and FAS were up regulated while FASL, c-FLIP, NMI, SOCS-1 and -3 (other known targets of STAT-5 activation [14]) were unaffected suggesting that in this *in vitro *model rIL-2 promotes the expression of genes associated with proliferation either directly (cyclins) or through an autocrine proliferative response (IL-2Rα) while decreases the responsiveness of cells to pro-apoptotic signals or other regulatory feedback mechanisms (SOCS-1 and -3). One putative target of STAT signaling is the interferon regulatory factor (IRF)-1 [46]. IRF-1 was induced by rIL-2 in PBMC (Figure 1C) as previously described [47] and its expression was most prominent in CD4 T cells stimulated in the absence of bystander PBMC (see later). IRF-1 is essential for cytotoxic T cell and natural killer cell function *in vivo *[48] and we previously observed that IRF-1 is consistently expressed together with several ISGs in melanoma metastases of patients receiving rIL-2 [24]. In addition, IRF-1 expression persists after treatment selectively in melanoma metastases undergoing complete regression in response to rIL-2 therapy combined to antigen-specific immunization [26]. Since IRF-1 expression is also induced by IFN-γ but not other type I IFNs [49] it remains to be elucidated whether this is a direct effect of rIL-2 on immune cells or depends upon the secondary production of IFN-γ.

##### Pathways associated with the common γ chain (γc) cytoplasmic tail

**Figure 2:**
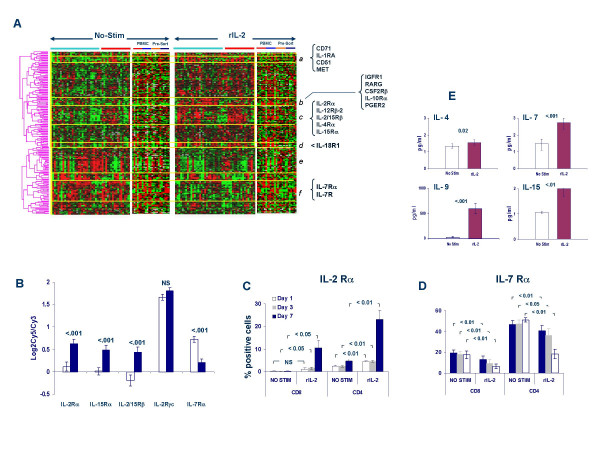
**A **– Clusterogram of genes associated with receptor function significantly modulated by rIL-2 obtained by pair wise *t *test. Light blue and red horizontal bares underline PBMC sample from Caucasian and Chinese donors; in separate panels CD4 (small red horizontal bar) and CD8 T cells (light blue bar) exposed to rIL-2 in the presence of PBMC and subsequently isolated are compared with CD4 and CD8 T cells (orange and dark blue horizontal bars respectively) purified before exposure to rIL-2. Distinct signatures are labeled in italic; **B **– mRNA expression of different sub-units of the IL-2R and the IL-7R without (white bars) or following stimulation (blue filled bars) with 300 IU/ml of rIL-2. Significance refers to a pair wise *t *test. **C **– Expression of the IL-2Ra chain in CD4 and CD8 T cells stimulated with rIL-2 in the presence of bystander PBMC (average ± SEM of six independent experiments, p2-values refer to a paired two-tailed *t *test between samples exposed to rIL-2 or non stimulated); **D **– Expression of the IL-7Ra chain in CD4 and CD8 T cells stimulated with rIL-2 in the presence of bystander PBMC (average ± SEM of six independent experiments, p2-values refer to a paired two-tailed *t *test between samples exposed to rIL-2 or non stimulated); **E **Concentration IL-4, IL-7, IL-9 and IL-15 (pg/ml, average of 47 donor samples) in the supernatant of PBMC cultures containing (rIL-2) or not containing (No Stim) 300 IU/ml of rIL-2. Supernatants were harvested after 24 hours. Statistical significance refers to a pair wise *t *test;

The γc chain has a relatively minor role in IL-2-depedent signaling. However, a central role of JAK-3 has been clearly demonstrated [50]. The expression of the γc chain was not significantly modulated by rIL-2 at the transcriptional level (Figure 2A and 2B). It is possible that JAK-3 over expression may serve as an enhancer of signaling through this IL-2R subunit. Interestingly, Calpain 1, a cysteine protease which down regulates γc chain expression by cleavage, was significantly up regulated by rIL-2 suggesting a possible regulatory mechanism [51]. Among other IL-2 dependent signaling events [14], the expression of src-family tyrosine kinases, the tyrosine kinase Pyk2 and the SH3- and ITAM containing molecules STAM and STAM2 was not significantly altered by rIL-2, However, the expression of STAM binding protein (STAMB) was strongly down regulated by rIL-2.

The activation of most of the genes associated with IL-2R signaling was strongly dependent upon the presence of the whole PBMC population. Analysis of T cells subsets (Figure 1B) clearly demonstrated that most genes were up-regulated in CD4 and CD8 T cells isolated from PBMC after exposure to rIL-2 but not in pre-sorted CD4 and CD8 subsets. Thus, we observed throughout the study that potentiation of rIL-2 signaling and activation of pro-proliferative (cyclins) and anti-apoptotic (BCL-2) signals depends upon the crosstalk between T cells and other "bystander" immune cells.

#### 2) Amplification of IL-2 signaling through enhancement of IL-2R complex affinity/availability and other receptors

The expression of several cytokine and growth factor receptors was altered by rIL-2. Four major self organizing gene clusters were up regulated (***a-d***, Figure 2A) and two down regulated (***e-f***, Figure 2A) by rIL-2.

Cluster *a *contained receptors associated with immune cell survival. The transferrin receptor, CD71 which is vital for continued cell growth, is induced in hematopoietic cells by various cytokines including IL-2 to maintain T cell proliferation [52,53] and its pattern of expression coincides with that of CD25 (IL-Rα) in T cells stimulated through CD3 and CD28 triggering [54]. The transferrin receptor is necessary for activated T cell survival and its blockage with monoclonal antibodies can arrest proliferation [55]. The induction of CD71 expression and its stabilization by IL-2 had been previously reported [56]. Surprisingly, the expression of CD71 was tightly linked to that of MET (receptor for hepatocite growth factor and the macrophage-stimulating protein), the urokinase plasminogen activator (PLAU) and its receptor (PLAUR) both required for activation of hepatocite growth factor-mediated cell proliferation [57]. This cluster also included CKCL16 which binds to CXCR6 and is involved in trafficking of immune cells to site of inflammation [58] and the interleukin-1 receptor antagonist which inhibits the pro-inflammatory effects of IL-1 [59]. The induction of expression of these receptors by rIL-2 was likely to occur in cells other than T cells since their expression was not significantly altered in CD4 and CD8 subsets whether stimulated in the presence or absence of bystander PBMC.

Cluster ***b ***included the insulin-like growth factor receptor-1 and the colony stimulating factor-2 receptor-β. The former has been associated with T cell activation/proliferation and sensitization to insulin [60]. This small cluster included also genes associated with down-regulation of T cell reactivity such as the prostaglandin E receptor [61], the retinoic acid receptor-γ (RARG) [62] and the IL-10Rα. Modulation of the last two receptors was T cell-specific because occurred in CD4 and CD8 T cells stimulated in the presence or absence of bystander PBMC.

Cluster ***c ***was characterized by the coordinate up regulation of several receptors for cytokines belonging to the common cytokine-receptor γ-chain (γc) (IL-2Rα, IL-2/IL-15Rβ, L-4Rα and-15Rα) and other immune stimulatory cytokines (IL-12Rβ and the IL18R accessory protein (Figure 2A and 2B). The expression of the IL-21R β-subunit which also participates to the cytokine-receptor common γ-chain complex [63] was not affected by rIL-2 stimulation. This is relevant since IL-21 participates in the modulation of lymphoid proliferation, apoptosis and differentiation. Unfortunately, no cDNA clones representative of the IL-9R (another common cytokine-receptor γc family member) were present in the arrays. Coordinately expressed with these receptors was also the expression of various nuclear receptors, integrin α 4 (CD49D, integral part of the VLA-4 receptor), the TCR δ chain, the platelet-derived growth factor receptor-α and several tumor necrosis factor receptor-associated transcripts). This observation suggests that rIL-2 amplifies its effects not only by co-regulating its own receptors but also by increasing the expression of receptors for other immune regulatory cytokines. Consistent with others' reports [39,64]., however, the three cDNA clones specific for the IL-7Rα chain (another member of the common cytokine-receptor γc chain family) were strongly and consistently down regulated (**Cluster *f***, Figure 2A and 2B). This is important because this cytokine regulates different phases of immune activation through differential expression of its receptor [65-67]. Interestingly, IL-7Rα down regulation was not associated with a decrease in the expression of BCL-2 or lung Kruppel-like factor, both involved in IL-7-dependent cell survival [68,69]. In fact, the expression of both genes was significantly up-regulated by rIL-2 supporting the hypothesis that rIL-2 (and indirectly IL-15) promotes clonal expansion of CD8+ T cells independently of IL-7 in the early phases of activation while it may not be involved in the maintenance of memory T cells which requires both IL-7Rα and IL-15Rα expression. This is also supported by the significant down-regulation of CD62L observed in rIL-2-stimulated PBMC (paired Student *t *test p_2_-value <0.001) compatible with an effector memory T cell phenotype [70,71]. The significance of the complete shut down of IL-7R transcription warrants more detailed analysis in the future since it was not as clearly seen in T cell subsets stimulated in the absence of PBMC. This suggests that this important modulation is dependent upon the influence of other immune cells present in the PBMC population. Subset analysis also demonstrated that the IL-2Rα chain was preferentially up regulated in CD4 compared with CD8 cells at this rIL-2 concentration. As for several other genes, cyto-fluorimetric analysis demonstrated that the changes in IL-2Rα (Figure 2C) and IL-7Ra (Figure 2D) progressed incrementally in time (3 to 7 days) underlying the lag existing between transcriptional activation and functional effect.

At the protein level, rIL-2 induced a significant release of cytokines belonging to the common cytokine-receptor γc chain family including IL-4, IL-7, IL-9 and IL-15 (Figure 2E). Obviously, IL-2 levels were also significantly increased in culture by it was not possible to distinguish whether endogenous expression of IL-2 by PBMC contributed to this increase. Thus, rIL-2 induces a generalized release of cytokines belonging to the common cytokine-receptor γc chain family but modulates cell responsiveness though the differential expression of cytokine-specific sub-units of the IL-2R. The significant modulation of different subunits of receptors associated to the common γc was not associated with modulation of the γc subunit itself suggesting that cytokine responsiveness within the family relies primarily on the differential expression of private cytokine-specific receptor subunits. Lack of modulation of the γc subunit of the IL-2R contrasts others experimental conditions in which IL-2 rendered T cells susceptible to apoptotic cell death through down-regulation of the γc in mice *in vivo *[72] underlining the strong dependence of cytokine signaling studies on the experimental condition tested.

#### 3) Modulation of TCR signaling

**Figure 3:**
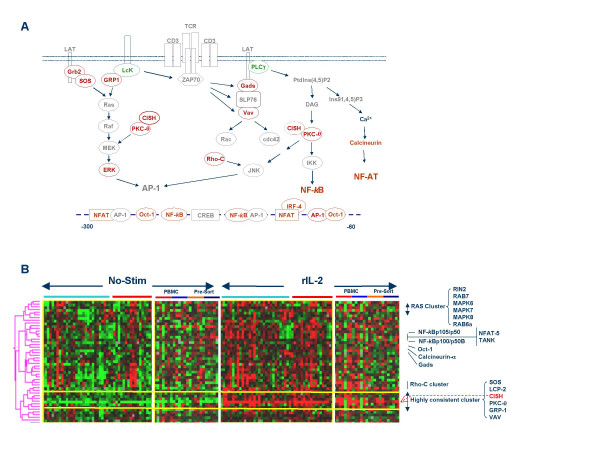
**A **– Signaling pathways downstream if TCR and CD28 as exemplified by modulation of IL-2 expression in T helper cells according to Gaffen SL and Liu KD [14]. In gray are genes whose expression was not significantly affected by rIL-2, in green those significantly down-regulated and in red those significantly up-regulated. **B **– Clusterogram of genes shown in panel A. Light blue and red horizontal bares underline PBMC sample from Caucasian and Chinese donors; in separate panels CD4 (small red horizontal bar) and CD8 T cells (light blue bar) exposed to rIL-2 in the presence of PBMC and subsequently isolated are compared with CD4 and CD8 T cells (orange and dark blue horizontal bars respectively) purified before exposure to rIL-2. The dashed orange arrow points at the expression of CISH in the clusterogram. Ratios are displayed according to the central method for display using a normalization factor [92].

**Figure 4:**
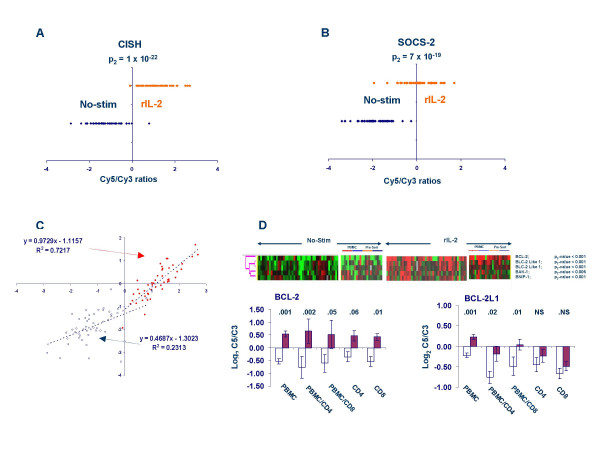
Relative expression (Log_2 _CY5/Cy3 ratio) of CISH (**A**), SOCS-2 (**B**) and scatter plot of their expression in baseline conditions (blue circles) and in response to rIL-2 (red circles) (**C**). Relative expression of BCL-2 and related genes (D); the p_2_-values refer to a two-tailed paired *t *test. Light blue and red horizontal bars underline PBMC sample from Caucasian and Chinese donors; in separate panels CD4 (small red horizontal bar) and CD8 T cells (light blue bar) exposed to rIL-2 in the presence of PBMC and subsequently isolated are compared with CD4 and CD8 T cells (orange and dark blue horizontal bars respectively) purified before exposure to rIL-2. Ratios are displayed according to the central method for display using a normalization factor [92]. The average expression of BCL-2 and BCL-2L1 is shown of PBMC and individual T cell subsets in the graph below.

The expression of IL-2 and other cytokines is controlled at multiple levels by T cells and it is mutually linked to TCR signaling [73,74]. Neither the TCR nor its associated co-receptors (CD3, ZAP-70, CD28 and CTLA-4) were affected by rIL-2. However, rIL-2 significantly affected downstream modulators of TCR signaling [14] converging toward cytokine promoter domains. For instance, the phospholipase C (PLC)γ-dependent pathway [75,76]. activates three major classes of transcription factors: NFAT, NF-*k*B and AP-1. In turn, rIL-2 can modify the expression of genes associated with these pathways (Figure 3A). The immediate signaling down-stream of linker for the activation of T cells (LAT) was consistently down-regulated (LcK, PLCγ) while the spleen tyrosine kinase (SYK) associated with TCR signaling was not significantly induced by rIL-2 (data not shown). However, downstream activators of cytokine transcription were up regulated. Ras associated GTPases expression was up-regulated in association with several MAP kinases (AMPK6, 7, 8). Tightly co-up regulated in the same cluster with NF-*k*B were several cDNA clones representing TANK (TRAF family member associated with NF-*k*B activation), NFAT-5 and Oct-1. Rho-C and associated genes were co-expressed in a separate cluster (Figure 3B). No induction of expression of either Jun or Fos (AP-1 complex) was observed (data not shown). A very interesting cluster included genes most consistently induced by rIL-2 in all donors (**between yellow horizontal bars**, Figure 3B). This cluster was enriched with genes classically associated with TCR signaling such as PKC-θ, SOS, GRP-1, and VAV. Particularly interesting was the up-regulation of CISH (cytokine inducible SH-2 domain containing protein) (paired *t *test p_2_-value 1 × 10^-23^). This was by far the gene most consistently up-regulated by rIL-2. CISH is a member of the SOCS adaptor family associated with TCR-mediated proliferation and survival of T cells [77] and its expression is specifically dependent upon the activation of STAT-5).)[45]. Increased expression of CISH has been reported in association with MAP kinase activation following TCR triggering leading to proliferative responses and prolonged survival of activated T cells [28,77]. CISH is a known early responder to IL-2 stimulation [78] and the high consistency of its induction suggests a crucial role of CISH in the interface between TCR-dependent and cytokine-dependent T cell proliferation and survival [28]. CISH and SOCS-2 were the only two members of the SOCS family to be significantly modulated by rIL-2 (Figure 4A and 4B). The expression of these two genes correlated loosely in resting PBMC (R^2 ^value = 0.23; **blue empty circles, **Figure 4C) but was tightly correlated following rIL-2 stimulation (R^2 ^value = 0.72; **red full circles, **Figure 4C). The same correlation was described by Li S *et al*. [77] in response to TCR stimulation suggesting overlapping biology and function of these two members of the SOCS family. Of interest, however, is the distinct behavior of STAT-5 in rIL-2 stimulated PBMC compared with TRC stimulated T cells. While in our study, IL-2 significantly induced the expression of STAT-5A, LI S *et al*. [77] did not observe induction of expression of STAT-5 mRNA following TCR stimulation although phospho-activation of the protein was observed. Thus, it is possible that rIL-2 enhances the down-stream signaling of TCR stimulation not only by allowing the activation of STAT-5 but also by enhancing its expression. However, since no correlation was noted, in our study, between STAT-5 expression and that of CISH or SOCS-2 (data not shown) it is possible that CISH modulation of TCR and IL-2 signaling is STAT-5 independent in the early phases of the response to rIL-2 as suggested by others [77]. CISH expression may be central to TCR-dependent signaling as it may function as an intermediate regulator transmitting signals from the TCR to the MAP kinase pathway through regulation of protein kinase-C (PKC) θ and subsequent activation of c-Jun-N-terminal kinase (JNK, TCR/CD28 dependent pathway) and ERK (TCR-dependent pathway) [77,79]. It is possible that the IL-2-induced enhancement of the expression of genes associated with the JNK pathway might be at the basis of the reduced dependence of T cells on CD28-mediated co-stimulation in presence of this cytokine. It is also not clear what the biological effects of SOCS-2 could be in response to rIL-2 stimulation. It was recently reported that SOCS-2 may participate in enhancing IL-2 signaling by accelerating the degradation of SOCS-3 [80]. Thus, rIL-2 induces in the conditions described here only two genes belonging to the SOCS family both of them possibly associated with stimulatory effects on T cell activation. Interestingly, contrary to others' reports [29], we did not identify significant alterations in the expression of SOCS-1. Subsets analysis again suggested that with few exceptions (i.e. CISH and Rho-C) the up-regulation of these signaling pathways was strongly dependent upon bystander PBMC as primarily isolated CD4 and CD8 cultures were not sensitive to this modulation (Figure 3B).

**Figure 5:**
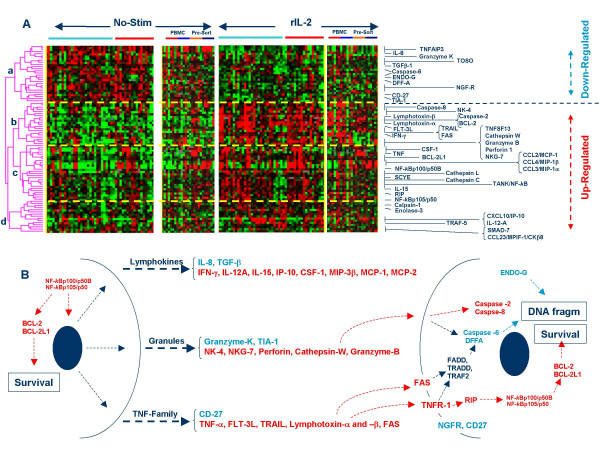
*A *– Clusterogram of genes coding for immune effector molecules including lymphokines, lytic granules and TNF family members. Only genes whose expression is significantly altered by rIL-2 (paired *t *test p_2_-value < 0.001) are shown. Light blue and red horizontal bares underline PBMC sample from Caucasian and Chinese donors; in separate panels CD4 (small red horizontal bar) and CD8 T cells (light blue bar) exposed to rIL-2 in the presence of PBMC and subsequently isolated are compared with CD4 and CD8 T cells (orange and dark blue horizontal bars respectively) purified before exposure to rIL-2. Four predominant nodes are underlined: node a) included genes down-regulated by rIL-2; node b) enriched with genes associated with lytic pathways; node c) enriched with cytokine genes and associated NF-*k*B pathways and node d) including a less characteristic mixture of genes. Horizontal blue bars above the clusterogram denote Caucasian and horizontal red bars Chinese subjects. Ratios are displayed according to the central method for display using a normalization factor [92]. *B *– Cartoon postulating the effector pathways affected by rIL-2 treatment; in light blue are genes whose expression is down regulated and in red genes whose expression is up-regulated. In dark blue are presented genes whose expression is not significantly affected by rIL-2. This cartoon represents an oversimplification of the various pathways and we refer the reader to the text for further details.

IRF-4 expression was strongly up regulated by rIL-2 (Figure 1C) confirming previous observations [39]. Expression of this transcription factor is specific to lymphoid and myeloid cells and combines adjacent to NFAT binding motives on cytokine promoter regions interacting with NFATc1 to enhance the production of IL-2, IL-4, IL-10 and IL-13 [81]. It has been shown that IRF-4 expression is driven through activation of NF-*k*B and NFAT pathways [82] and it is dependent upon antigen-mimetic stimuli such as TCR cross-linking or treatment with phorbol ester [83]. Interestingly, while IL-4 (Figure 2D) and IL-13 (Figure 5) were up-regulated by rIL-2 no change in the release of IL-10 was noted in this study either at mRNA or protein level.

#### 4) Lymphokine and immune-effector molecule regulation by rIL-2

**Figure 6:**
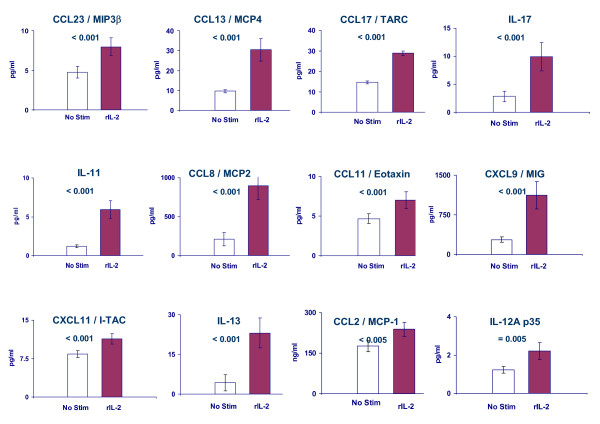
Average concentration of cytokines/chemokines (pg/ml) in the supernatant of PBMC cultures from 47 donors harvested 24 hours after stimulation with (rIL-2) or without (No Stim) rIL-2 (300 IU/ml). Statistical significance refers to a pair wise *t *test. Chemokine nomenclature is reported according to the IUIS/WHO subcommittee on chemokine nomenclature [121]. Cytokine/chemokines are displayed in order of significance. In addition to these cytokines/chemokines also IFN-γ (p_2_-value < 0.01), IFN-α (p_2_-value = 0.01), lymphotoxin-α (p_2_-value = 0.02), IL-5 (p_2_-value = 0.03) and CCL10/IP-10 (p_2_-value = 0.04) were significantly up-regulated though to a lower degree of significance.

Immune effector functions including lymphokines, lytic granule-associated molecules and TNF/TNFR family members were extensively modulated by rIL-2 (Figures 5 and 6). Four major clusters were identified. **Cluster *a ***included genes down-regulated such as IL-8 and TGF-β also reduced at the protein level in culture supernatants (data not shown). This is in contrast with our previous *in vivo *observation of consistently increased serum levels following systemic rIL-2 administration suggesting that the release of these cytokines is dependent upon interactions with cells other than PBMC [11,24]. Down regulation of these cytokines was specific to CD4 and CD8 cells independent of the presence of bystander PBMC. Consistent with others' reports [29,39]., several receptors associated with cell survival were also down-regulated including CD-27, the nerve growth factor receptor and TOSO. TIA-1 (a lytic granule associated protein whose over expression before rIL-2 therapy but not after has been linked to the immune responsiveness of melanoma metastases) was down-regulated in accordance to others' *in vitro *experimental findings [29]. Interestingly, down regulation of TIA-1 occurred only in CD4 and CD8 subsets exposed to rIL-2 in the absence of bystander PBMC suggesting that the regulation of this biomarker of responsiveness to rIL-2 is strongly dependent upon the immunological microenvironment [26]. **Cluster b) **included genes associated with lytic function such as cathepsins, granzyme-B, perforin 1, NK-4/IL32 and NKG7 which we have previously reported over expressed by *in vitro*-activated immunization-induced T cells [25]. Several of these genes were also found to be over expressed in a melanoma metastasis regressing in response to rIL-2 therapy [24]. Interestingly, this cluster also included IL-15, lymphotoxin-α and β, IFN-γ, FLT-3, caspase-2 and -8 and BCL-2. The expression of the genes included in this cluster was strictly dependent on rIL-2 stimulation as practically none of the non-stimulated samples expressed them at any level. CD4 and CD8 subsets stimulated in the presence of bystander PBMC behaved similarly with strong up regulation of all the genes included in cluster *b*. However, the induction was subset-specific in CD4 and CD8 cells exposed to rIL-2 in the absence of bystander cells. In particular, CD4 T cells over expressed caspase-8, lymphotoxin-α and β, FLT-3, and NK-4/IL-32 while the over expression of granzyme-B, perforin and NKG-7 became restricted to CD8 T cells (see also later). This information may be extremely important for future interpretation of *in vivo *data. For instance, as later discussed, NK-4/IL-32 is becoming increasingly recognized as a biomarker central to immune effector function. Its over expression in acutely inflamed organs may depend upon modulation by bystander cells.

**Cluster c) **included predominantly lymphokines (CSF-1, TNF-α, IL-15, SCYE, MCP-1, MIP-1α and MIP-1β) whose expression was coordinately associated with that of NF-*k*B and TANK [84]. This cluster was characterized by expression of genes preferentially expressed by CD4 and CD8 T cells in the absence of by-stander PBMC even in the absence of rIL-2 stimulation. Finally, **cluster d) **contained a functionally heterogeneous array of genes including IP-10 and IL-12A also found to be over-expressed at the protein level in culture supernatants (paired *t *test p_2_-value < 0.01 and = 0.005 respectively) as well as survival/apoptosis associated factors such as calpain-1, Enolase-3 and SMAD-7 [85,86].

#### 5) T cell subset-specific effects of rIL-2 stimulation

The transcriptional alterations induced by rIL-2 in PBMC were largely consistent with previous reports [29,39]. However, it remained to be elucidated to what degree rIL-2 acts directly on T cells rather than through bystander interactions. Therefore, we compared the transcriptional profile of CD4 and CD8-expressing T cell obtained from 6 donors (3 Chinese: D12, 15 and 36 and 3 Caucasian: D38, 43 and 45). T cell subsets were sorted by negative bead separation before (referred thereafter as CD4 or CD8 T cells) or after (referred here as PBMC/CD4 or PBMC/CD8) exposure to 300 IU/ml rIL-2 for 4 hours. Purity after separation was similar in all conditions and averaged 89.3% ± 0.9% (range 80% to 97%). This approach could segregate transcriptional alterations induced in CD4 and CD8 T cells by rIL-2 directly (CD4 and CD8 subsets) from those requiring the presence of PBMC (PBMC/CD4 or PBMC/CD8 subsets). The complete data set is available as additional file 3. All subsequent analyses were performed by comparing samples with a two-tailed paired or unpaired Student *t *test as appropriate. For the subset analyses the cut-off p_2_-value of significance was set at < 0.05 due to the smaller sample size as done by others [29,39].

##### Baseline differences between CD4 and CD8 T cells

**Figure 7:**
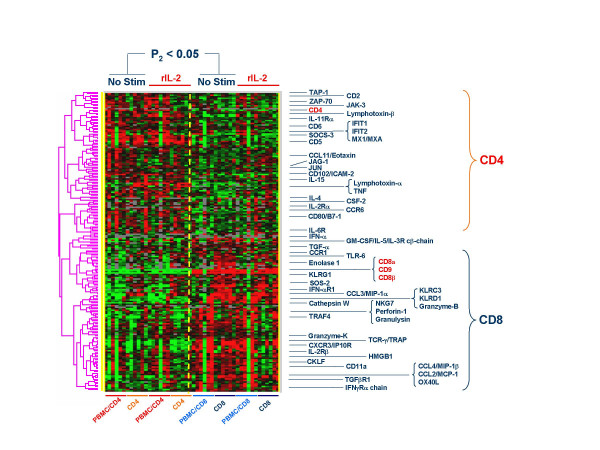
Baseline differences between the transcriptional profiles of CD4 and CD8-expressing T cells obtained from 3 Chinese (D12, 15 and 36) and 3 Caucasian (D38, 43 and 45) donors. T cell subsets were separated by negative bead separation (Miltenyi Biotech, Bergisch Gladbach, Germany) at the time of thawing before exposure to 300 IU/ml rIL-2 (referred here simply as CD4 or CD8 T cells, orange and dark blue horizontal bars respectively) or after *in vitro *culture with or without rIL-2 for four hours (referred here as PPBMC/CD4 or PBMC/CD8; red or light blue horizontal bars respectively). Analysis of significance is based on a paired two-tailed Student *t *test with a cut off p_2_-value < 0.05 comparing the combined CD4 and PBMC/CD4 samples to the respective CD8 and PBMC/CD8 samples not stimulated with rIL-2 (No Stim); Data from samples exposed to rIL-2 (rIL-2) are also shown. Selected genes with known immune function are shown in this clusterogram (see text for details). The dashed vertical line separated CD4 from CD8 T cell samples. Ratios are displayed according to the central method for display using a normalization factor [92].

Baseline differences between CD4 and CD8 T cells were first evaluated (paired *t *test p_2_-value < 0.05) comparing the combined PBMC/CD4 and CD4 samples to the respective PBMC/CD8 and CD8 samples not exposed to rIL-2. This analysis identified 1,078 cDNA clones differentially expressed (869 with annotated function). As expected, CD4 T cells consistently expressed CD4 (Figure 7). In addition, CD4 T cells expressed proportionally higher levels of genes associated with T cell signaling/activation (ZAP-70, JAK-3, AATF, Jun-b and JAG-1) and immune modulation (IL-4, IL-15, Lymphotoxin-α and -β, CCL11/Eotaxin, CSF-2 and TNF-α). The structurally and physically related accessory molecules CD5 and CD6 were preferentially expressed in CD4 T cells. This may be important since these co-receptors co-localize in the immunological synapsis to provide complementary accessory signals during T cell activation and/or differentiation [87]. CD8 T cells preferentially expressed CD8; CD9, a tetraspanin previously known to be selectively expressed by CD4(+)CD45RA(+) naïve T cells and involved in their activation [88] was coordinately expressed with CD8. This may be interesting since CD9 functions as an alternative receptor for the chemoattractant interleukin-16 whose primary ligand is CD4 [89]. It is possible that CD9 expressed by CD8 T cells might compensate for lack of CD4 allowing responsiveness to IL-16 during inflammation. CD8 expression was also tightly linked to several killer cell-like receptors (KLR) such as KLRG1 (an inhibitory C-type lectin expressed in natural killer and activated CD8 cells [90,91]. KLRC3 and KLRD1. In addition, CD8 T cells expressed proportionally higher levels of genes associated with effector functions including granzyme-B and K, perforin, NKG-7, granulysin and pro-inflammatory cytokines including IFN-α, CCL4/MIP-1β, CCL2/MCP-1 and OX40 Ligand. Of interest was the differential expression of the IL-2R subunits by CD4 and CD8 T cells. CD4 cells displayed higher expression of CD25 (IL-2Rα chain) while CD8 displayed higher levels of the IL-2/15Rβ chain (Figure 7). This pattern was recognized also at the protein level by FACS analysis which demonstrated that IL-2Rα expression was higher in CD4 then CD8 T (Figure 2C).

Importantly, rIL-2 exposure did not significantly change the proportional expression of most of these lineage-specific genes suggesting that T cell differentiation is not influenced by rIL-2. It is important to emphasize, however, that this analysis portrays proportional differences between CD4 and CD8 T cells according to a central method of normalization [92]. Thus, it should not be implied that: 1) higher expression by a subset corresponds to no expression by the other; 2) rIL-2 does not modulate the expression in a given subset.

##### Transcriptional changes consistently induced by rIL-2 in T cell subsets

**Figure 8:**
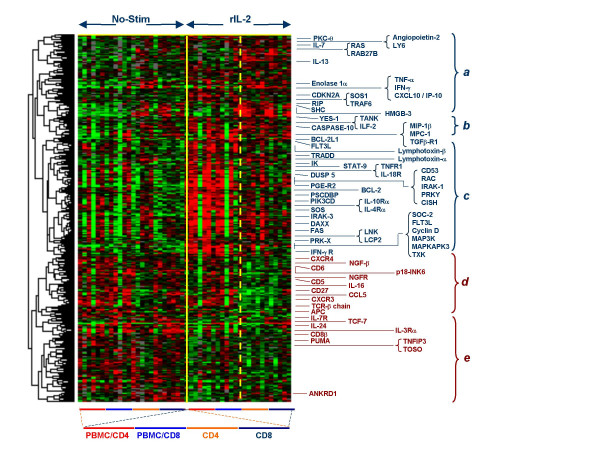
Genes differentially expressed by CD4 and CD8 cells upon *in vitro *exposure to rIL-2 (300 IU/ml) independent of the presence or absence of the rest of the PBMC population. In spite of significance in expression in all populations compared with baseline conditions, clusters could be identified with preferential expression according to the *in vitro *condition in which rIL-2 was administered. In blue are clusters of genes up regulated by rIL-2 (main clusters *a*, *b *and *c*) and in maroon those down regulated (clusters *d *and *e*).

We first identified genes consistently induced by rIL-2 in either CD4 or CD8 T cells independent of the presence of bystander PBMC by combining data from PBMC/CD4, CD4, PBMC/CD and CD8 samples. This analysis identified 717 clones (597 with known annotation) that predominantly recapitulated the finding observed in whole PBMC. However, their proportional expression was strongly biased according to stimulatory condition (Figure 8). Distinct patterns could be identified based on self organizing clusters: a) genes predominantly up-regulated by rIL-2 in the absence of PBMC. These genes included several cytokines such as IL-7, IL-13, TNF-α, IFN-γ and IP-10; b) genes similarly up regulated independently of condition of stimulation including MIP-1β, MCP-1 and Caspase-10; c) genes preferentially induced in the presence of PBMC. These included anti-apoptotic factors such as BCL-2 and BCL-2L1 and signaling molecules associated with T cell activation belonging to the MAP kinase pathway reinforcing the impression that T cell survival and activation is strongly dependent upon the presence of bystander cells. The same group also contained several cytokine receptor genes indicating that up-regulation of receptors follows pathways quite different from that of cytokine activation. A good example was the induction of the IFN-γ receptor in T cells stimulated in the presence of PBMC while IFN-γ was induced predominantly when T cells were stimulated individually; d) genes predominantly down regulated when T cells are exposed to rIL-2 in the absence of bystander PBMC. This group included cytokines such as IL-16 and IL-24 associated with important chemoattractant activity for T cells. In addition, this cluster included genes associated with T cells survival such as CD27, nerve growth factor (NFG) and several surface markers known to be down-regulated by rIL-2 [29], thus bystander PBMC may regulate some of the negative effects or rIL-2 on T cells; e) Genes down regulated by rIL-2 independent of stimulatory condition. These included several cytokine receptors such as IL-7R and IL-13Rα which were strongly down-regulated in contrast with the up-regulation of IL-7 and IL-13 by the same population of lymphocytes suggesting the production of these cytokines is meant for cells other than those receiving the rIL-2 stimulus. Interestingly, also the TCR-β chain and its co-receptor CD8 were down-regulated in this model.

##### Subset-specific changes

**Figure 9:**
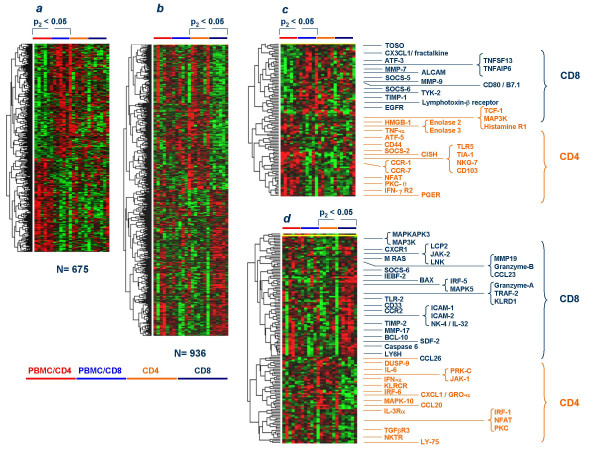
Genes differentially expressed by CD4 and CD8 cells upon *in vitro *exposure to rIL-2 (300 IU/ml) depending upon the presence or absence of the rest of the PBMC population (for labeling see Figure 6): *a*) genes differentially modulated between CD4 and CD8 cells that were stimulated in the whole PBMC population and subsequently separated (paired Student *t *test p_2_-value < 0.05);*b*) genes differentially modulated between CD4 and CD8 cells that were stimulated after being separated from the whole PBMC population by negative selection (paired Student *t *test p_2_-value <0.05); c) selected genes differentially modulated between CD4 and CD8 cells that were stimulated in the whole PBMC population and subsequently separated (from series in panel *a*) associated with T cell activation/effector function; c) selected genes differentially modulated between CD4 and CD8 cells that were stimulated after separation from the whole PBMC population by negative selection (from series in panel *b*) associated with T cell activation/effector function.

To better characterize subset-specific changes induced by rIL-2 we normalized the data set subtracting Log2Cy5/Cy3 ratios of non-stimulated samples from the corresponding values in samples exposed to rIL-2. A paired Student *t *test was applied to the normalized data set to identify differences between PBMC/CD4 and PBMC/CD8 (Figure 9A) or CD4 and CD8 T cells (Figure 9B). This analysis emphasizes the changes from baseline in the expression of genes rather than their absolute expression. The first analysis identified 675 cDNA clones (569 with annotated functions) differentiating rIL-2-exposed PBMC/CD4 from PBMC/CD8. The second analysis identified 936 cDNA clones (777 annotated) differentially expressed between rIL-2 exposed CD4 and CD8 T cells suggesting stronger functional differentiation in the absence of bystander effect. There was very little overlap of genes induced in CD4 and CD8 cells in the presence of absence of PBMC. Specialized function of CD4 and CD8 cells appeared to be dampened by rIL-2 stimulation in the presence of bystander effects. This could be exemplified by the analysis of a restricted number of genes with known effects on T cell activation and effector function. A striking example was the lack of differential induction of typical effector molecules such as Granzyme A and B and NK-4/IL-32 in CD8 T cells stimulated in the presence of bystander PBMC (Figure 9C). On the contrary typical functions commonly associated with CD4 or CD8 T cells were clearly present in CD4 and CD8 subsets exposed to rIL-2 in the absence of bystander PBMC (Figure 9D). In particular, while the granzymes, KLRD1 and NK4/IL-32 were typically up-regulated in CD8 cells, several cytokines such as CCL26/eotaxin-3, IL-6, IFN-α, CXCL1/GRO-α and CCL20/MIP3α were specifically expressed by CD4. Novel was the identification of the differential expression of IRF-5 over expressed in CD8 T cells compared to the over expression of IRF-1 and IRF-6 in CD4 T cells. In the absence of bystander effect CD8 cells expressed several chemokine receptors including CXCR1 and CCR2. The expression of CXCR1 is noteworthy considering that CD4 T cells expressed instead its ligand CXCL1/GROα suggesting a possible chemotactic activity of activated CD4 T cells on CD8 T cell. This analysis emphasized the intensification of specialized CD8 and CD4 functions in the absence of bystander effects while the presence of bystander PBMC resulted in a global enhancement in activation and survival signals. Thus, it appears that the transcriptional profile of T cell subsets follows a bipolar pattern whereby some molecular signatures are emphasized in the presence of bystander cells while others are emphasized in the absence of them.

## Discussion

The systemic effects of rIL-2 administration have been difficult to characterize due to the pleiotropism of this cytokine resulting from the cross talk of immune and other bystander cells [11,24]. *In vitro *human models and experimental animal models suggest contrasting properties of rIL-2 ranging from pro-inflammatory to regulatory effects. [7,15,16,26,93-95] Empirically, rIL-2 can induce dramatic regressions in about 15% of patients with advanced cancer [1,23]. representing a powerful human model to study the requirement(s) for tumor immune rejection and, more generally, the implementation of effective immune responses. We have argued that tools are available for high-throughput, hypothesis-generating investigations of such mechanism(s) through serial sampling of tumors before, during and after rIL-2 treatment [24,26,96,97]. In addition, insights on the mechanisms of action or rIL-2 in vivo might help understanding it possible role as an adjuvant for active-specific immunization efforts [98] or for the expansion of tumor-reactive T cells ex vivo [99]. Regrettably, practical restrictions have so far limited the broad exploitation of this strategy for a conclusive characterization of the biological signatures predictive of immune responsiveness. Anecdotal studies from our group, combined with review of the literature suggest that tumor immune responsiveness follows a pattern consistent with an immunological constant of rejection that applies also to acute allograft rejection [100,101], flares of autoimmune disease [102,103], clearance of virus during acute infections or tissue damage during chronic viral infection [104-106].

An indolent pattern of low grade inflammation is commonly observed in pathological conditions in which a chronic inflammatory status does not lead to resolution of the process by clearing its cause. This occurs in chronic viral infections, chronic allograft rejection well-controlled by mild immune suppression, lingering autoimmune reactions and cancer. In such cases, transcriptional profiling consistently identifies the activation of ISGs whose expression seems to be part of the inflammatory process but not sufficient for its clearance [26,90,100,104-106] Similarly, treatments inducing immune stimulation consistently induce ISGs independent of clinical outcome [24,107]. Clearance of the pathogenic process requires the incremental activation of additional immune effector functions related to cytotoxic function [24,26,90,100]. A recent prospective double-blinded study applying transcriptional analysis to basal cell carcinoma samples treated with a toll receptor 7 agonist at doses that consistently induce immune-mediated regression of lesions [108-111]. confirmed that the expression of ISGs is only part of a complex picture in which immune effector signatures predominate through the enhancement of acute inflammation and cytotoxic mechanisms (Panelli et al. in preparation).

Pending further *in vivo *clinical studies, we studied *in vitro *molecular patterns induced by rIL-2 that could provide a road map for the interpretation of future *in vivo *observations. PBMC were selected to approximate the lively cross talk occurring among immune cells *in vivo*. For this reason, we elected not to use cycloheximide to block protein synthesis to discriminate the direct effects of rIL-2 from those induced secondarily through autocrine or bystander intermediaries. Information about the immediately/early-regulated genes by rIL-2 is already available through an elegant analysis in which such parameters were carefully controlled [39]. This study was primarily aimed at mimicking the interactive effects of rIL-2 in the host. A limitation of this model is the absence of other important cells involved in the cross talk such as specialized immune cells sequestered in immune organs, endothelial cells etc. An example of such limitation was the expression of IL-8 significantly down-regulated *in vitro *(Figure 6) but consistently up regulated at the transcriptional [24] and protein levels [11] in blood samples obtained from patients receiving systemic rIL-2 therapy. Thus, an obvious conclusion of this study is that the effects of rIL-2 are strictly dependent upon the immunological environment in which the cytokine is received. Monocytes and macrophages have long been known to play a key role in T cell activation through the production of cytokines such as IL-1, IL-6, IL-12, IL-18 and TNF-α or through direct cell-cell interactions [39,112]. Interestingly, IL-1R, IL-12-R and IL-18R were all up-regulated by rIL-2 in PBMC and in CD4 and CD8 subsets exposed to rIL-2 in the presence of bystander PBMC suggesting that these accessory signals are strongly enhanced only in the presence of additional immune cells. In addition, T cell interactions may influence each other. This might explain the paradoxical observation that regulatory T cells may be increased in frequency when rIL-2 is administered to cancer patients with a normal immune system [7] while lymphodepletion may enhance the immune effectiveness of this cytokine upon removal of regulatory mechanisms [8]. Indeed, the activation of some effector cytotoxic T cell function was more appreciable when CD8 expressing T cells were exposed to rIL-2 in the absence of bystander PBMC (Figure 5A, Figure 8 and Figure 9D).

Microarray analysis and other high throughput technologies have been previously utilized to identify novel IL-2 target genes [29,39,113]. However, the analyses were performed on a relatively small number of individuals of homogeneous background. This limited the power of the analysis to a relatively high threshold of false discovery (p-value ≤ 05). Even with this relatively lax threshold only 460 genes were found to be consistently up-regulated and 419 down-regulated by rIL-2 exposure for a total of 879 differentially expressed genes in one study [29] and a total of 316 in another [39]. The present study expanded these observations surveying 47 donors of two ethnic backgrounds (Chinese and Caucasian) allowing the identification of 1,690 genes differentially expressed at a 10-fold more stringent threshold (paired two-tailed Student *t *test p_2_-value < 0.005). The data almost perfectly matched previous descriptions [29,39]. For instance 14 of 19 genes identified by an Affymetrix U95Av2 platform as regulated by rIL-2 were found to be concordantly altered in expression with a high degree of significance (p_2_-value <0.001) by our study, four where not included in our cDNA platform and only two did not match the described results [39]. Similarly, strong concordance was observed with subsequent observations based on transcriptional analyses performed on Affymetrix platforms [29,39]. This is remarkable because in other's experimental conditions PBMC or T cells had been pre-activated for 72 hours with anti-CD3 antibody to induce IL-2R expression and T cell activation [29,39]. suggesting that at the rIL-2 concentrations used here pre-activation of T cells is not necessary to induce rL-2 responsiveness. This is an important detail because the concentrations used in this study are likely to exceed those present in physiological conditions but are close or even below those achieved during systemic rIL-2 administration with therapeutic purposes [24]. However, some important differences were noted in our model compared with the previous studies that combined rIL-2 stimulation with T cell pre-activation by anti-CD3 or anti-CD28 mAbs [29,39]. For instance, among genes involved in feedback regulation of IL-2 signaling, we found that several dual specificity phosphatases (DUSP) were altered in expression including DUSP-5 but not DUSP-6 as reported by others [29] (Figure 1A). In addition, we did not observe up-regulation of SOCS-1 while SOCS-2 was dramatically up-regulated. This is diametrically opposite to others' observation suggesting that these pathways may be strongly dependent upon T cell pre-activation. Since SOCS-2, opposite to SOCS-1 has protective effects on T cell survival [80] it may explain why T cells that had not been pre-activated through TCR-associated signaling may be least prone to activation-induced cell death [16]. This also is reflected by the lack of effects of rIL-2 on FasL expression which is required for activation-induced cell death [114] and it is induced by rIL-2 in T cells pre-activated through TCR triggering [29].

Genetic differences have been shown to be associated with distinct pathologies [32]. However, genetic background did not appear to affect significantly the response to rIL-2 although borderline differences where observed between the two ethnic groups. Moreover, a polymorphism of the IL-2Rβ chain (asp→glu, 391) adjacent to a tyrosine residue that serves at docking site for STAT signaling was relatively conservative and observed only in heterozygous conditions. Comparison of PBMC bearing the mutant allele (relatively more frequent in the Chinese population) did not predict particular patterns of gene expression. Thus, it could be safely concluded that the response to rIL-2 is genetically conserved and ethnic differences between these two populations are unlikely to affect significantly the individual response to rIL-2. This is important because it is unclear to what degree genetic background is responsible for the broad range of individual immune responsiveness and susceptibility to the toxic effects of rIL-2. This study did not identify dramatic differences directly related to rIL-2 signaling that could account for such variability.

Transcriptional patterns induced by rIL-2 where found to be quite consistent in PBMC largely overlapping those observed in T cell subsets stimulated in the presence of by-stander PBMC. In particular, gene functions associated with T cell survival and proliferation were induced similarly in CD4 and CD8 T cells stimulated in the presence of bystander PBMC suggesting that cytokine or cell-to-cell interactions may be important for T cell survival in either subset. This is consistent with previous reports [29]. In contrast, several functions specific to CD4 and CD8 T cells remained unaltered or where accentuated when T cells were exposed to rIL-2 in the absence of bystander PBMC suggesting that rIL-2 directly nurtures specialized T cell functions. In particular, CD8 T cell activation toward a natural killer phenotype was clearly fostered by rIL-2 in the absence of bystander PBMC (Figure 8). This may be important because the *in vivo *conditions either in the circulation or within the tumor microenvironment may most often approximate the presence of bystander PBMC. Transcriptional patterns of melanoma metastases biopsied three hours after rIL-2 administration consistently lack pattern suggestive of CD8 effector gene activation [24]. Interestingly, ISGs were equally induced by rIL-2 in PBMC and T cell subsets (data not shown) as a most consistent pattern associated with the activity of this cytokine *in vitro *and *in vivo *[24] although it bears little relevance to the implementation of immune effector function [24,100,102,104].

In summary, this study suggests that the predominant effect of rIL-2 administration at the doses used in this study is stimulatory to T cells with induction of proliferation, maintenance of survival and activation of effector functions while regulatory mechanisms associated with induction of apoptosis, suppression of cytokine signaling were not observed during these early phases of PBMC stimulation. This is consistent with others' observations in PBMC and purified CD4 and CD8 T cells that could not readily explain the survival versus death-inducing functions of rIL-2 based on the kinetics of gene induction [29]. Moreover, presence of bystander PBMC appears to dampen the effector function of CD4 and CD8 subsets, a finding that might explain the recent clinical observation of the enhanced efficacy of rIL-2 based therapies in the context of lymphodepletion [8].

## Materials and methods

### Donor's characteristics and PBMC collection

Specimens were obtained from normal donors at the Department of Transfusion Medicine, National Institutes of Health (NIH) under an institutionally approved protocol (04-CC-0007). All donors signed an institutionally approved informed consent. PBMC were collected by apheresis in the DTM Research Clinic using the standard DTM operating procedures and the Fenwal CS3000 blood cell separator. PBMC were isolated by Ficoll gradient separation and frozen immediately in 1 × 10^8 ^cells/vial aliquots until analysis.

### *In vitro *stimulation

After thawing, PBMC were placed at 1×10^7 ^cells per well in 6 well plate (Costar Cambridge, MA) using OPTI-MEM-without serum supplementation and were incubated at 37°C overnight. Next day, rIL2 (300 IU/ml) was added to the cells. As suggested by others [29,39]. and according to our previous experience [24] the stimulation was carried for 4 hours. After 4 hour adherent and non-adherent cells were removed from each well and centrifuged. An additional set of experiments was performed by stimulating in identical conditions PBMC from 6 donors (3 Caucasian, donors 38, 43 and 45 and 3 Chinese, donors 12, 15 and 36). In these experiments CD4 and CD8 subsets were separated from PBMC after the 4 hour stimulation period using negative selection (Miltenyi Biotech, Bergisch Gladbach, Germany) as previously described [25] at 4°C to prevent RNA metabolism/degradation. In parallel, PBMC from the same donors were subjected to negative separation for CD4 and CD8 selection. The two subpopulations were then stimulated with rIL-2 following identical procedure.

### Supernatant collection and protein analysis platform

Supernatants from cell cultures were obtained 24 hours after stimulation with rIL-2 (300 IU/ml) or from parallel cultures in which no rIL-2 had been supplemented. The supernatants were immediately stored in cryogenic vials (Nunc cat# 363401, St Pleasant Prairie, WI) in 1 ml/vial aliquots, snap frozen in dry ice and stored at -80°C. Protein levels was assesses on multiplex protein-based platform (Pierce SearchLight Proteome Arrays, Boston, MA) as previously described [11] covering a total of 80 soluble factors. Data are presented as pg/ml)

### RNA preparation, amplification and labeling

Total RNA from test PBMC from normal donors was extracted and amplified into anti-sense RNA as previously described (aRNA) [115])[24,116]. Total RNA from PBMC pooled from six normal Caucasian individuals not part of the present protocol was extracted and amplified into aRNA to serve as constant reference [115])[24]. Test and reference RNA were labeled with Cy5 (red) and Cy3 (green) respectively and co-hybridized to a custom-made17.5 K cDNA (UniGene cluster) micro-array. Micro-arrays were printed at the Immunogenetics Section, DTM, CC, NIH with a configuration of 32 × 24 × 23 and contained 17,500 elements. Clones used for printing included a combination of the Research Genetics RG_HsKG_031901 8 k clone set and 9,000 clones selected from the RG_Hs_seq_ver_070700 40 k clone set. The 17,500 spots included 12,072 uniquely named genes, 875 duplicated genes and about 4,000 expression sequence tags.

#### Flow cytometry

PBMC from leukocyte aphaeresis of 6 donors were thawed and plated in complete Iscove medium (Life Technologies, Grand Island, NY) supplemented with 10% heat inactivated human AB serum, 10 mM HEPES buffer, 100 U/ml penicillin-streptomycin, 0.5 mg/ml amphotericin B and 0.03% glutamine, at the density of 10^6 cells/well in 48 multiwell plate. After resting overnight half of the wells were treated with human recombinant IL-2 300 U/ml (rHuIL-2, Chiron Co, Emeryville, CA). IL-2 was added every two days. Treated and untreated cells were harvested after 1–3–7 days. The modulation of the expression of CD25 (IL-2 receptor alpha chain) and CD127 (IL-7 receptor) were tested by staining the cells with the following antibodies: CD4-APC, CD8-PE or CD8-FITC, CD3-PerCP and CD25-FITC (all from BD Biosciences Pharmingen, San Diego, CA) or CD127-PE (Beckman Coulter, Fullerton, CA). As negative control cells were stained with IgG FITC or PE conjugated, according with the antibody's isotype. Cells were analyzed with FACS sort (BD Bioscience) gathering them on living lymphocytes CD3 positive.

#### ILRβ sequencing

Total RNAs were extracted from each donor's PBMC as previous described and converted to cDNA by reverse transcription. PCR was performed by using iProof High-Fidelity DNA polymerase (Bio-Rad) according to the manufacturer protocol.

The following primers were used in the PCR reaction: forward: 5'-TGCCACCGCCCCATGTCTCA-3' reverse: 5'-CACAAAGATGGTACACACGGATCATT-3' using the following conditions:

98° 30 seconds, 98° 10 seconds, 62° 30 seconds, 72° 2 minutes for 30 cycles, 72° 10 minutes for final extension. PCR products were purified by adding 3 μl ExoSAP-IT (USB) per 20 μl of PCR product, at 37° for 15 minutes followed by incubation at 80° 15 minutes to inactivate the enzyme. The sequencing reaction was set up using the BigDye Terminator v3.1 Cycle Sequencing kit and ABI Prism 3700 DNA Analyzer.

The following primers were used for sequencing:

F1: 5'-TGCCACCGCCCCATGTCTCA-3'

F2: 5'-CCATCCAGGACTTCAAGCC -3'

F3: 5'-AGGACAAGGTGCCTGAGC-3'

F4:5'-TGGTGCTGCGAGAGGCTG-3'

F5: 5'-CAGCCTGAGCGTGCTTTC-3'

F6: 5'CCTGCTGCATCTTCCCACA-3'

F7: 5'TCTGACCAGCAGCCTATGAG-3'

R1: 5'-CGAACTCCAGGTGTCTTTCAA-3'

R2: 5'-CTCTATCTCCAAGGCATCCG-3'

R3: 5'-AACAGGGTCCTTCTGAGGCT-3'

R4: 5'-GGAATAGCATGTGCAACAGAG-3'

R5: 5'-GTCAGAGTTAGCTGGGACTGG-3'

R6: 5'-GGATAAGGAGACCGACTTGC-3'

### Statistical analysis

The raw data were filtered to exclude spots with minimum intensity by arbitrarily setting a minimum intensity requirement of 300 in both fluorescence channels. If the fluorescence intensity of one channel was over and that of the other below 300 the fluorescence of the low intensity channel was arbitrarily set to 300. Spots with diameters <25 μm and flagged spots were excluded from the analysis. The filtered data were then normalized using the lowess smother correction method. All statistical analyses were performed using the log_2_-based ratios normalizing the normal value in the array equal to zero.

Validation and reproducibility were measured using an internal reference concordance system based on the expectation that results obtained through the hybridization of the same test and reference material in different experiments should perfectly collimate. The level of concordance was measured by periodically re-hybridizing the melanoma cell line A375-melanoma (American Type Culture Collection, Rockville MD) to the reference samples consisting of pooled PBMC as previously described [117]. This analysis demonstrated a higher than 95% concordance level. Non-concordant genes were excluded from subsequent analysis.

Principal component analysis (PCA) was performed using Partek array analysis software (Partek Inc., St Charles, Missouri) over the entire data set. Supervised class comparison utilized the BRB ArrayTool [118] developed at NCI, Biometric Research Branch, Division of Cancer Treatment and Diagnosis. Paired samples (i.e. unstimulated and rIL-2 stimulated PBMC from the same donors) were compared with a two-tailed paired Student *t *test. Unpaired samples were tested with a two-tailed un-paired Student *t *test assuming unequal variance. All analyses were tested for a univariate significance threshold set at a p_2_-value <0.005. Gene clusters identified by the univariate *t *test were challenged with two alternative additional tests, a univariate permutation test (PT) and a global multivariate PT. The multivariate PT was calibrated to restrict the false discovery rate to 10%. Genes identified by univariate *t *test as differentially expressed (p_2_-value < 0.005) and a PT significance <0.05 were considered truly differentially expressed. Gene function was assigned based on Database for Annotation, Visualization and Integrated Discovery (DAVID) [119] and Genontology [118].

## Abbreviations

Caucasian, **Cau**; Cytokine inducible SH-2 domain containing protein, **CISH**; Chinese, **Chi**; dual-specificity phosphatases, **DUSP**; interferon, **IFN**; interferon regulatory factor, **IRF**; interferon stimulated genes, **ISGs**; interleukin, **IL**; interleukin-X receptor, **IL-**X **R**; Janus kinase, **JAK**; c-Jun-N-terminal kinase, **JNK**; killer cell-like receptor, **KLR**; linker for the activation of T cells, **LAT**; natural killer transcript 4, **NK4/IL32**; nuclear factor of activated T cells, **NFAT**; octameric binding transcription factor, **Oct**; phosphatidylinositol 3-kinase, **PI 3-K**; peripheral blood mononuclear cells, **PBMC**; protein kinase C, **PKC**; recombinant interleukin-2, **rIL-2**; retinoic acid receptor-γ, **RARG**; signal transducer and activator of transcription, **STAT**; spleen tyrosine kinase, **SYK**; suppressor of cytokine signaling, **SOCS**; T cell receptor, **TCR**; TRAF family members associated with NF-kB activation, **TANK**.

## Supplementary Material

Additional file 1 Genes differentially expressed between non-treated and rIL-2 treated PBMC from 47 donors; 1,690 genes were identified to be significantly differentially expressed at a paired *t *test cut-off p_2_-value < 0.005 (see Table 1). Click here for file

Additional file 2 Sequence information about the IL-2Rβ chain in the 47 donors. Click here for file

Additional file 3 Transcriptional alterations induced in CD4 and CD8 T cells by rIL-2 in the presence or absence of PBMC at a paired *t *test cut-off p_2_-value < 0.005. Click here for file
