# Supplementary material for: Molecular signatures induced by interleukin-2 on peripheral blood mononuclear cells and T cell subsets
Source: J Transl Med. 2006 Jun 28;4:26. doi: 10.1186/1479-5876-4-26 (PMC1557669; doi:10.1186/1479-5876-4-26)
Supplement: Additional file 2 — Sequence information about the IL-2Rβ chain in the 47 donors. [file 1479-5876-4-26-S2.doc]

|  | N.T.Position | 881 | 1283 | 1304 | 2202 | 2617 | 2677 | 2976 | 3597 |
| --- | --- | --- | --- | --- | --- | --- | --- | --- | --- |
|  | dbSNP # | rs2228142 | new | rs228942 | rs228941 |  |  |  |  |
|  | Reference | C/C | C/C | C/C | G/G | T/T | A/A | T/T | G/G |
|  | A.A.position | 250 | 384 | 391 |  |  |  |  |  |
|  | Function | syn | syn | nonsyn | Un-trans | Un-trans | Un-trans | Un-trans | Un-trans |
|  | A.A.Change |  |  | C-Asp, A-Glu |  |  |  |  |  |
| Donor # | RACE |  |  |  |  |  |  |  |  |
| 3 | Chinese | T/C | C/C | A/C | G/G | T/T | A/A | T/T | G/G |
| 6 | Caucasian | T/C | C/C | A/C | G/C | T/T | A/A | T/C | G/G |
| 9 | Caucasian | T/T | C/C | A/C | G/C | T/T | A/A | T/T | A/G |
| 10 | Chinese | T/T | C/C | A/C | G/C | T/T | A/A | T/T | G/G |
| 11 | Caucasian | T/T | C/C | A/C | G/C | T/T | A/A | T/T | A/G |
| 12 | Chinese | T/T | C/C | A/C | C/C | T/T | A/A | T/T | G/G |
| 14 | Chinese | C/C | C/C | A/C | G/C | T/T | A/A | T/T | G/G |
| 16 | Caucasian | T/T | C/C | A/C | G/C | T/T | A/A | T/T | G/G |
| 17 | Chinese | C/C | C/C | A/C | G/C | T/T | A/A | T/T | G/G |
| 19 | Caucasian | T/T | C/C | A/C | C/C | T/T | A/A | T/T | G/G |
| 21 | Chinese | T/C | C/C | A/C | G/C | T/T | A/A | T/T | G/G |
| 26 | Caucasian | T/C | C/C | A/C | G/C | T/T | A/A | T/T | G/G |
| 27 | Chinese | T/T | C/C | A/C | C/C | T/T | A/A | T/T | G/G |
| 38 | Caucasian | T/T | C/C | A/C | G/C | T/T | A/A | T/T | G/G |
| 39 | Chinese | T/C | C/C | A/C | G/C | T/T | A/A | T/T | G/G |
| 43 | Caucasian | T/C | C/C | A/C | G/C | T/T | A/A | T/T | G/G |
| 44 | Chinese | T/C | C/C | A/C | G/C | T/T | A/A | T/T | G/G |
| 47 | Caucasian | T/T | C/C | A/C | G/G | T/T | A/A | T/T | G/G |
| 1 | Caucasian | T/C | C/C | C/C | G/G | T/T | A/A | T/T | A/G |
| 2 | Caucasian | C/C | C/C | C/C | G/C | T/T | A/A | T/T | G/G |
| 4 | Chinese | C/C | C/C | C/C | G/G | T/T | A/A | T/T | G/G |
| 5 | Chinese | T/C | C/C | C/C | G/C | T/T | A/A | T/T | G/G |
| 7 | Caucasian | T/T | C/C | C/C | G/G | T/T | A/A | T/T | A/A |
| 8 | Caucasian | C/C | C/C | C/C | G/G | T/T | A/A | T/T | G/G |
| 13 | Chinese | C/C | C/C | C/C | G/G | T/T | A/A | T/T | G/G |
| 15 | Chinese | T/C | C/C | C/C | G/G | T/T | A/A | T/T | G/G |
| 18 | Caucasian | C/C | C/C | C/C | G/G | T/T | A/A | T/T | G/G |
| 20 | Caucasian | C/C | C/C | C/C | G/G | T/T | A/A | T/T | G/G |
| 22 | Caucasian | T/T | C/C | C/C | G/G | T/T | A/A | T/T | G/G |
| 23 | Chinese | C/C | C/C | C/C | G/G | T/T | A/A | T/T | G/G |
| 24 | Caucasian | T/C | C/C | C/C | G/C | T/T | A/A | T/T | G/G |
| 25 | Caucasian | T/C | C/C | C/C | G/G | T/T | A/A | T/T | G/G |
| 28 | Caucasian | C/C | C/C | C/C | G/G | T/T | A/A | T/T | G/G |
| 29 | Chinese | C/C | C/C | C/C | G/G | T/T | A/A | T/T | G/G |
| 30 | Caucasian | T/C | C/T | C/C | G/C | T/T | A/A | T/T | G/G |
| 31 | Caucasian | T/C | C/C | C/C | G/C | T/T | A/G | T/T | G/G |
| 32 | Caucasian | T/C | C/C | C/C | G/G | T/T | A/A | T/T | G/G |
| 33 | Caucasian | C/C | C/C | C/C | G/G | T/T | A/A | T/T | G/G |
| 34 | Caucasian | T/C | C/C | C/C | G/C | A/T | A/A | T/T | G/G |
| 35 | Caucasian | T/T | C/C | C/C | G/G | T/T | A/A | T/T | G/G |
| 36 | Chinese | C/C | C/C | C/C | G/G | T/T | A/A | T/T | G/G |
| 37 | Caucasian | T/T | C/C | C/C | G/G | T/T | A/A | T/T | G/G |
| 40 | Chinese | T/T | C/C | C/C | G/C | T/T | A/A | T/T | G/G |
| 41 | Caucasian | C/C | C/C | C/C | G/G | T/T | A/A | T/T | G/G |
| 42 | Caucasian | C/C | C/C | C/C | G/G | T/T | A/A | T/T | G/G |
| 45 | Caucasian | C/C | C/C | C/C | G/G | T/T | A/A | T/T | G/G |
| 46 | Caucasian | C/C | C/C | C/C | G/C | T/T | A/A | T/T | G/G |
